# Synthesis and *in Vitro* Antitumor Activity of a Novel Series of 2-Pyrazoline Derivatives Bearing the 4-Aryloxy-7-chloroquinoline Fragment

**DOI:** 10.3390/molecules191118656

**Published:** 2014-11-14

**Authors:** Alba Montoya, Jairo Quiroga, Rodrigo Abonia, Manuel Nogueras, Justo Cobo, Braulio Insuasty

**Affiliations:** 1 Heterocyclic Compounds Research Group, Department of Chemistry, Universidad del Valle, Apartado Aéreo 25360, Colombia; E-Mails: montoyaarias340@gmail.com (A.M.); jaiquir@gmail.com (J.Q.); rodrigo.abonia@correounivalle.edu.co (R.A.); 2 Department of Inorganic and Organic Chemistry, Universidad de Jaén, Jaén 23071, Spain; E-Mails: mmontiel@ujaen.es (M.N.); jcobo@ujaen.es (J.C.)

**Keywords:** microwave irradiation, Claisen-Schmidt condensation, chalcones, cyclocondensation reaction, 2-pyrazolines, antitumor activity

## Abstract

A new series of *NH*-pyrazoline derivatives **6** was synthesized by cyclocondensation reaction of novel [(7-chloroquinolin-4-yl)oxy]chalcones **5** with hydrazine hydrate. The treatment of pyrazolines **6** with acetic anhydride or formic acid yielded the *N*-acetyl- or *N*-formylpyrazoline derivatives **7**–**8**, respectively. These novel 2-pyrazoline derivatives **6**–**8** were evaluated by the U.S. National Cancer Institute (NCI). Compounds **7b**,**d**,**f** and **8c**,**f** showed remarkable antitumor activity against 58 cancer cell lines, with the most important GI_50_ values from *in vitro* assays ranging from 0.48 to 1.66 μM. The 2-pyrazoline derivatives bearing the 4-aryloxy-7-chloroquinoline fragment are thus considered to be useful leads for the rational design of new antitumor agents.

## 1. Introduction

**Figure 1:**
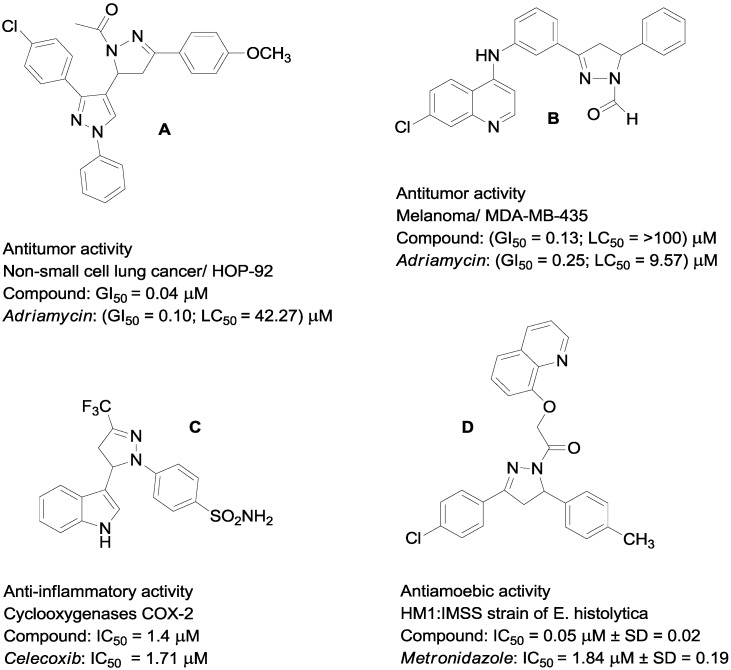
Some pyrazolines with remarkable biological activity.

The identification of novel structures that can be potentially useful in designing new, potent selective and less toxic anticancer agents is still a major challenge for medicinal chemistry researchers [1]. It is well known that many natural or synthetic chalcones are highly active in a large pharmaceutical and medicinal applications [2,3]. Several strategies for the synthesis of these systems based on formation of carbon-carbon new bonds have been reported and among them the direct Aldol and Claisen-Schmidt condensations still occupy prominent position [4]. Chalcones are found to be effective as antimicrobial [5], antiviral [6], cardiovascular [7] and anti-inflammatory [8] agents; as well as their recognized synthetic utility. After the pioneering works of Fischer and Knoevenagel in the late nineteenth century [9], the reaction of α,β-unsaturated aldehydes and ketones with hydrazines became one of the most popular method for the preparation of 2-pyrazolines, which have attracted interest due to their diverse biological activities such as antitumor, immunosuppressive, antibacterial, anti-inflammatory, anticancer, antidiabetic and antidepressants [1,10,11,12,13,14,15,16]. Among the existing various pyrazoline type derivatives, 1-acetylpyrazolines have been identified as one of the most promising scaffolds, which were found to display fungicidal and insecticidal activities [17]. Examples of such systems are shown in Figure 1.

On the other hand, the quinoline motive occurs in several natural compounds (cinchona alkaloids) and pharmacologically active substances displaying a broad range of biological activity [18]. In recent years it have been reported that the incorporation of these active pharmacophores in the structure of new heterocyclic compounds could potentiate their biological activity [19,20]. Prompted by the above mentioned biological properties of chalcones, pyrazolines and the additional value of having quinoline motives in their structures and in continuation with our current studies directed toward the synthesis of novel nitrogen containing heterocyclic compounds with biological activity [21,22,23,24,25,26], we have decided to explore a series of new pyrazolines containing the 4-aryloxy-7-chloroquinoline fragment in their structures derived from chalcones as starting materials. The results discussed in this paper reflect our efforts in discovering new potential anticancer chemotherapeutic agents.

## 2. Results and Discussion

### 2.1. Chemistry

**Scheme 1:**
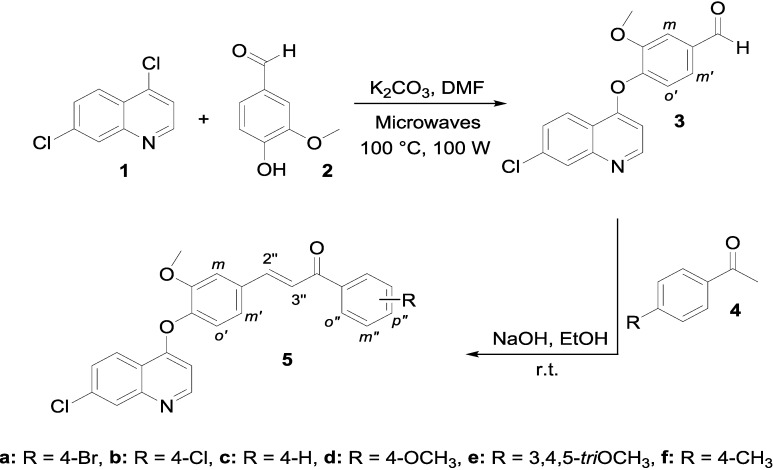
Synthesis of novel [(7-chloroquinolin-4-yl)oxy]chalcones **5**.

In order to obtain the new key chalcone derivatives **5** as starting materials for the synthesis of the target products **6**–**8**, the synthesis of the precursor 4-(7-chloroquinolin-4-yloxy)-3-methoxybenzaldehyde (**3**) was performed by the selective nucleophilic aromatic substitution (S_N_Ar) of the 4-chlorine atom on 4,7-dichloroquinoline (**1**) with 4-hydroxy-3-methoxybenzaldehyde (**2**). This S_N_Ar process was carried out by microwave irradiation of the reagents for 6 min at a power of 100 W and temperature of 100 °C. The present protocol is quite convenient and environmentally friendly, since the reaction proceeds under mild reaction conditions when compared to classical methods [27]. Then the Claisen-Schmidt condensation of precursor **3** with several aromatic acetophenones led to the formation of **5** in good to excellent yields (58%–95%) (Scheme 1 and Experimental Section).

The Claisen-Schmidt condensation was conducted in ethanol at room temperature, using drops of 20% sodium hydroxide solution as catalyst. The IR spectrum of compound **5a**, for example, showed a characteristic absorption band at 1662 cm^−1^ corresponding to the stretching vibration of the carbonyl group. Two doublets at 7.81 and 7.45 ppm with *J* = 15.7 Hz which correspond to protons H-2'' and H-3'' were observed in the ^1^H-NMR spectrum of compound **5a**, confirming the *E*-configuration for the double bond of the α,β-unsaturated carbonyl moiety.

**Scheme 2:**
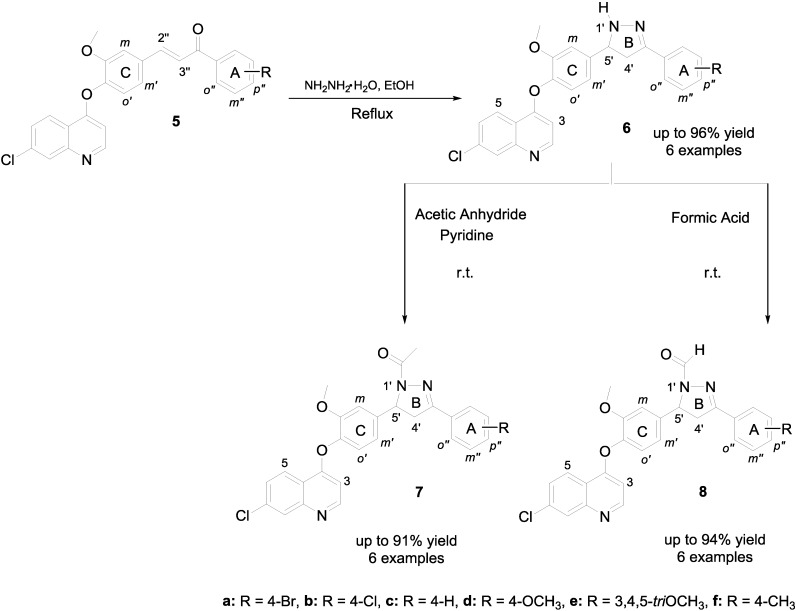
Synthesis of new *NH*, *N*-acetyl and *N*-formylpyrazolines **6**–**8**.

Chalcones **5** were reacted with hydrazine hydrate, heating to reflux in EtOH, in order to accomplish the synthesis of the *NH*-pyrazolines **6** (Scheme 2), which were obtained in acceptable to excellent yields (71%–96%). Substitution on *N*-1 of pyrazolines **6** was carried out by treating either with acetic anhydride or with formic acid under stirring at room temperature for 10–30 min, to afford the novel *N*-acetyl- or *N*-formylpyrazoline derivatives **7**–**8** respectively (Scheme 2). These new pyrazolines **6**–**8**, were fully characterized by means of spectroscopic techniques such as FT-IR, ^1^H-NMR, ^13^C-NMR and MS (see Experimental Section). As an example, in the IR spectrum of compound **8b**, an absorption band is observed at 1,674 cm^−1^ which corresponds to the stretching vibration of the C=O amide functionality and a broad stretching band for the C=N and C=C functionalities is observed at 1591 cm^−1^. In the ^1^H-NMR spectrum the protons on the diastereotopic center C-4', of the pyrazoline ring appears as two double-doublets at δ = 3.33 and 3.98 ppm with ^2^*J*_AM_ = 18.2, ^3^*J*_AX_ = 5.1 and ^3^*J*_MX_ = 11.6 Hz, while the H-5' proton is observed as a double-doublet at 5.64 ppm with ^3^*J*_MX_ = 11.6 and ^3^*J*_AX_ = 5.1 Hz. All carbon atoms were completely assigned using DEPT-135, HSQC and HMBC techniques. Finally, mass spectra of compounds **6**–**8** showed also well-defined molecular ions.

### 2.2. Anticancer Activity

As a preliminary screening, structures of all new compounds (*i.e.*, **6**–**8** series) were submitted to the Developmental Therapeutics Program (DTP) at National Cancer Institute (NCI) for evaluation of their anticancer activity against different human tumor cell lines. Thirteen of the submitted structures (*i.e.*, **6b**–**e**; **7b**,**d**,**e**,**f** and **8b**–**f**) were selected and subjected to the preliminary evaluation against the 58 tumor cell lines at a single dose of 10 μM after 48 h of incubation. The output from the single dose screening was reported as a mean graph available for analysis by the COMPARE program (data are not shown). The results of this first assay showed that compounds **7b**,**d**,**f** and **8c**,**f** were active. Then, active compounds passed to a second stage in order to determine their cytostatic activity against the 58 tumor cell lines represented in *leukemia*, *melanoma*, *lung*, *colon*, *brain*, *breast*, *ovary*, *kidney* and *prostate* panels; where the testing results were expressed according to the following three parameters: GI_50_ which is the molar concentration of the compounds required to inhibit the growing of the cell lines to 50% (relative to untreated cells). TGI as the molar concentration that causes total growth inhibition, and LC_50_ which is a parameter of cytotoxicity and reflects the molar concentration needed to kill 50% of the cells [28]. The active compounds were evaluated at five concentration levels (100, 10, 1.0, 0.1, and 0.01 μM) and the test consisted of a 48 h continuous drug exposure protocol using sulforhodamide B (SRB) protein assay to estimate cell growth. Details of this evaluation method, and the complementary information related with the activity pattern over all cell lines, have been published [29,30,31,32,33]. As an outstanding result, compounds **7b**,**d**,**f** and **8c**,**f** exhibited remarkable activities, with GI_50_ ranges from 10^−7^ to 10^−6^ M, nevertheless, a raw comparison of the activities of our obtained compounds **6**–**8** with respect to the activity reported for the standard drug adriamycin, used by NCI as control, reflects that the activities displayed for our compounds were lower than for the standard drug control as follows: compounds **7d**, **7f** and **8f** displayed activities with GI_50_ values of 1.66, 0.48 and 1.13 × 10^−6^ M respectively, against the SNB-75 cell line (*CNS Cancer* panel), while this value was 0.07 × 10^−6^ M for the standard drug adriamycin; compound **7b** displayed GI_50_ value of 1.40 × 10^−6^ M against BT-549 (*breast cancer* panel), while the value against the same cell line for adriamycin was 0.23 × 10^−6^ M; finally the compound **8c** displayed GI_50_ value of 1.50 × 10^−6^ M against HOP-92 (*non-small cell lung* panel), while the value was 0.10 × 10^−6^ M for the standard drug adriamycin. The above results suggest that the compounds **7b**,**d**,**f** and **8c**,**f** are promising structures, of the obtained compounds, for our future drug development antitumor studies. On the other hand, the cytotoxicity associated with the latter compounds, measured as LC_50_ are around 100 μM, for most cell lines, indicating a low toxicity of such compounds for normal human cell lines as required for potential antitumor agents (see Table 1).

**Table 1:** *In vitro* testing expressed as growth inhibition of cancer cell lines for compounds **7b**,**d**,**f** and **8c**,**f** and the standard drug adriamycin **^a^**.

Panel/Cell Line	Compounds									Standard Drug		
	7b		7d		7f		8c		8f	Doxorubicin (Adriamycin), NSC 123127 ^d^		
	GI_50_^b^ (µM)	LC_50_^c^ (µM)	GI_50_^b^ (µM)	LC_50_^c^ (µM)	GI_50_^b^ (µM)	LC_50_^c^ (µM)	GI_50_^b^ (µM)	LC_50_^c^ (µM)	GI_50_^b^ (µM)	LC_50_^c^ (µM)	GI_50_^b^ (µM)	LC_50_^c^ (µM)
***Leukemia***												
CCRF-CEM	>100	>100	>100	>100	>100	>100		>100	>50	>50	0.08	100.00
HL-60(TB)	>100	>100	>100	>100	>100	>100		>100	>50	>50	0.12	89.33
K-562	>100	>100		>100	>100	>100		>100	>50	>50	0.19	100.00
MOLT-4	>100	>100	>100	>100	>100	>100		>100	>50	>50	0.03	100.00
RPMI-8226	>100	>100	>100	>100	>100	>100		>100	>50	>50	0.08	100.00
SR	>100	>100	>100	>100	>100	>100	>100	>100	>50	>50	0.03	100.00
***Non-Small Cell Lung***												
A549/ATCC		>100		>100		>100	8.87	>100	>50	>50	0.06	100.00
EKVX		>100	51.7	>100	>100	>100	3.67	>100	7.75	>50	0.41	47.97
HOP-62	2.36	>100	3.37	>100	4.30	>100	12.4	>100	1.68	>50	0.07	67.61
HOP-92	2.32	>100	3.03	>100	30.1	>100	1.50	>100	9.10	>50	0.10	42.27
NCI-H226	2.47	>100	2.78	>100	5.38	>100	5.24	>100	1.81	>50	0.05	6.40
NCI-H23		>100	11.9	>100	2.71	>100	4.82	>100	6.93	>50	0.15	13.15
NCI-H460		>100		>100		>100	18.5	>100	>50	>50	0.02	51.29
NCI-H522		>100	7.57	>100	>100	>100	5.28	>100	2.72	>50	0.03	2.80
***Colon Cancer***												
COLO 205		>100		>100	>100	>100	>100	>100	>50	>50	0.18	4.33
HCC-2998	>100	>100	>100	>100	>100	>100	>100	>100	>50	>50	0.26	21.68
HCT-116		>100	2.61	>100	0.68	>100	6.07	>100	2.50	>50	0.08	54.58
HCT-15	>100	>100	>100	>100	>100	>100	>100	>100	>50	>50	6.46	100.00
HT29		>100		>100	>100	>100	>100	>100	>50	>50	0.12	67.45
KM12	>100	>100	>100	>100	>100	>100	>100	>100	>50	>50	0.27	92.68
SW-620	>100	>100	>100	>100	>100	>100	>100	>100	>50	>50	0.09	58.61
***CNS Cancer***												
SF-268		>100	9.28	>100	38.4	>100	23.2	>100	11.3	>50	0.10	30.48
SF-295	4.22	>100	5.02	>100	2.52	>100	5.58	>100	4.41	>50	0.10	69.98
SF-539	2.58	>100	2.40	>100	11.7	>100	10.3	>100	3.85	>50	0.12	27.23
SNB-19	>100	>100	26.8	>100	37.4	>100	26.6	>100	9.53	>50	0.04	49.77
SNB-75	1.66	>100	1.66	48.6	0.48	>100	3.31	>100	1.13	38.8	0.07	3.30
U251	3.09	>100	4.51	>100	1.41	>100	19.8	>100	6.50	>50	0.04	30.62
***Melanoma***												
LOX IMVI		>100		>100		>100	>100	>100	>50	>50	0.07	50.35
MALME-3M	>100	>100		>100	>100	>100		>100	>50	>50	0.12	3.97
M14	>100	>100	93.0	>100	>100	>100	5.83	>100	14.2	>50	0.18	4.05
MDA-MB-435	>100	>100	>100	>100	>100	>100		>100	>50	>50	0.25	9.57
SK-MEL-2	15.9	>100	7.13	90.9	15.8	>100	8.68	>100	7.39	45.2	0.17	1.06
SK-MEL-28		>100		>100	>100	>100	>100	>100	>50	>50	0.21	15.92
SK-MEL-5	>100	>100	>100	>100	>100	>100	2.18	>100	>50	>50	0.08	0.49
UACC-257	>100	>100		>100	>100	>100		>100	>50	>50	0.14	8.15
UACC-62	>100	>100	5.35	>100	>100	>100	6.76	>100	11.2	>50	0.12	0.74
***Ovarian Cancer***												
IGROV1	>100	>100	6.29	>100	38.2	>100	35.0	>100	24.8	>50	0.17	100.00
OVCAR-3		>100	5.40	>100	>100	>100	18.6	>100	>50	>50	0.39	84.33
OVCAR-4		>100		>100		>100	4.17	>100		>50	0.37	74.30
OVCAR-5	>100	>100	>100	>100	>100	>100	>100	>100	>50	>50	0.41	100.00
OVCAR-8		>100	3.63	>100		>100	4.91	>100		>50	0.10	43.25
NCI/ADR-RES		>100		>100		>100	3.23	>100	>50	>50	7.16	100.00
SK-OV-3		>100	6.01	>100	32.4	>100	4.56	>100	7.20	>50	0.22	100.00
***Renal Cancer***												
786-0	2.63	>100	4.52	>100	1.17	>100	11.5	>100	5.07	>50	0.13	51.64
A498	4.09	>100	15.2	>100	13.1	>100	6.90	>100	5.73	>50	0.10	1.90
ACHN		>100	4.03	>100	2.76	>100	17.4	>100	4.39	>50	0.08	100.00
CAKI-1	>100 3.32	>100	>100	>100		>100	5.20	>100	>50	>50	0.95	100.00
RXF 393	>100	>100	5.04	>100	3.57	>100	15.4	>100	4.16	>50	0.10	4.69
SN12C	2.63	>100	>100	>100	>100	>100	>100	>100	>50	>50	0.07	72.44
UO-31		>100	>100	>100		>100	13.5	>100	>50	>50	0.49	26.18
***Prostate Cancer***												
PC-3		>100	-	-	>100	>100	2.80	>100	>50	>50	0.32	87.10
DU-145	>100	>100	>100	>100	>100	>100	>100	>100	>50	>50	0.11	100.00
***Breast Cancer***												
MCF7	>100	>100	29.4	>100	>100	>100	5.70	>100	>50	>50	0.03	51.29
MDA-MB-231/ATCC		>100	7.52	>100	11.3	>100	28.5	>100	4.23	>50	0.51	34.75
HS 578T	2.94	>100	5.15	>100	2.60	>100	5.60	>100	1.82	>50	0.33	85.70
BT-549	1.40	>100	4.00	>100	3.48	>100	2.24	>100	3.96	>50	0.23	21.33
T-47D	2.85	>100	5.54	>100	2.28	>100	10.3	>100	2.90	>50	0.06	85.70
MDA-MB-468	4.22	>100	7.43	>100	20.7	>100	2.17	>100	2.59	>50	0.05	2.52

^a^ Data obtained from NCI’s *in vitro* disease-oriented human tumor cell lines screen [27,28,29,31,32]; ^b^ GI_50_ was the drug concentration resulting in a 50% reduction in the net protein increase (as measured by SRB staining) in control cells during the drug incubation. Determined at five concentration levels (100, 10, 1.0, 0.1, and 0.01 μM); ^c^ LC_50_ is a parameter of citotoxicity and reflects the molar concentration needed to kill 50% of the cells; ^d^ The values of activity against human tumor cell lines displayed by adriamycin correspond to the reported by NCI at highest concentration of 10^−4^ M.

## 3. Experimental Section

### 3.1. General Information

Commercially available starting materials, reagents and solvents were used as supplied. Microwave reactions were performed in glass vessels (10 mL) using a CEM Discover Focused Microwave Synthesis System^TM^ apparatus, with power output from 0 to 300 W. TLC analyses were performed on Merck silica gel 60 F254 aluminum plates. Melting points were determined in a Büchi melting point apparatus and are uncorrected. IR spectra were performed on a Shimadzu FTIR 8400 spectrophotometer in KBr disks. The ^1^H- and ^13^C-NMR spectra were run on a Bruker DPX 400 spectrophotometer operating at 400 MHz and 100 MHz, respectively, using chloroform-*d* and dimethylsulfoxide-*d*_6_ as solvents and tetramethylsilane as internal reference. The mass spectra were obtained on a Hewlett Packard HP Engine-5989 spectrometer (equipped with a direct inlet probe) operating at 70 eV. The elemental analyses were obtained using a Thermo-Finnigan Flash EA1112 CHN (Elemental Microanalysis Ltd, Devon, UK) elemental analyzer.

### 3.2. Chemistry

#### 3.2.1. General Procedure for the Synthesis of Compound **3** under Microwave Irradiation

A mixture of 4,7-dichloroquinoline **1** (0.5 g, 2.5 mmol), vanillin **2** (0.38 g, 2.5 mmol), potassium carbonate (1 g, 7.2 mmol) in *N*,*N*-dimethylformamide was submitted to microwave irradiation for 6 min at a power of 100 W and a temperature of 100 °C. The reaction mixture was cooled and cold water was added. The precipitate of *4-[(7-Chloroquinolin-4-yl)oxy]-3-methoxybenzaldehyde* (**3**) formed was filtered and recrystallized from ethanol. Beige solid; 80% yield; mp: 140–142 °C. FTIR ʋ (cm^−1^): 1701 (C=O), 1591 and 1563 (C=N and C=C) ^1^H-NMR (CDCl_3_) δ ppm 3.82 (s, 3H, OCH_3_), 6.43 (d, *J* = 5.2 Hz, 1H, H-3), 7.32 (d, *J* = 8.0 Hz, 1H, H*o*'), 7.53 (dd, *J* = 9.0, 2.0 Hz, 1H, H-6), 7.55 (dd, *J* = 8.0, 1.6 Hz, 1H, H*m*'), 7.59 (d, *J* = 1.6 Hz, 1H, H*m*), 8.09 (d, *J* = 2.0 Hz, 1H, H-8), 8.30 (d, *J* = 9.0 Hz, 1H, H-5), 8.65 (d, *J* = 5.2 Hz, 1H, H-2), 9.99 (s, 1H, CHO). ^13^C-NMR (CDCl_3_) δ ppm 56.1, 104.1, 111.6, 119.5, 122.8, 123.4, 125.2, 127.3, 128.1, 135.2, 136.3, 147.6, 150.3, 152.1, 152.3, 160.9, 190.7. MS (70 eV) *m/z* (%): 313 (84, M^+^), 197 (99), 176 (100), 162 (87), 135 (43), 99 (54). Anal. Calcd. For C_17_H_12_ClNO_3_: C, 65.08; H, 3.86; N, 4.46. Found: C, 64.98; H, 3.89; N, 4.41.

#### 3.2.2. General Procedure for the Synthesis of Chalcones **5a**–**f**

A mixture of aldehyde **3** (300 mg, 1 mmol), the appropriate acetophenone **4** (1 mmol), 20% aq NaOH (0.8 mL) and 95% EtOH (30 mL) was stirred at room temperature for 2 h. The solid formed was filtered and washed with ethanol. No further purification was needed and products were used such as were obtained.

*(E)-1-(4-Bromophenyl)-3-[4-((7-chloroquinolin-4-yl)oxy)-3-methoxyphenyl]prop-2-en-1-one* (**5a**). White solid; 93% yield; mp: 177–179 °C. FTIR ʋ (cm^−1^): 1662 (C=O), 1605 and 1585 (C=N and C=C). ^1^H-NMR (CDCl_3_) δ ppm 3.82 (s, 3H, OCH_3_), 6.43 (d, *J* = 5.3 Hz, 1H, H-3), 7.22 (d, *J* = 8.2 Hz, 1H, H*o*'), 7.28 (d, *J* = 1.6 Hz, 1H, H*m*), 7.34 (dd, *J* = 8.2, 1.6 Hz, 1H, H*m*'), 7.45 (d, *J* = 15.7, 1H, =CH), 7.53 (dd, *J* = 8.9, 2.0 Hz, 1H, H-6), 7.65 (d, *J* = 8.5 Hz, 2H, H*o*''), 7.81 (d, *J* = 15.7, 1H, =CH), 7.89 (d, *J* = 8.5 Hz, 2H, H*m*''), 8.08 (d, *J* = 2.0 Hz, 1H, H-8), 8.33 (d, *J* = 8.9 Hz, 1H, H-5), 8.64 (d, *J* = 5.3 Hz, 1H, H-2). ^13^C-NMR (CDCl_3_) δ ppm 56.1, 103.6, 112.7, 119.5, 122.1, 123.3, 123.6, 127.1, 128.0, 128.2, 128.7, 130.2, 132.0, 133.8, 136.2, 136.8, 144.2, 144.3, 150.2, 151.9, 152.3, 161.4, 189.2. MS (70 eV) *m/z* (%): 493 (75, M^+^), 495 (100), 414 (46), 315 (33), 183 (40), 160 (45). Anal. Calcd. For C_25_H_17_BrClNO_3_: C, 60.69; H, 3.46; N, 2.83. Found: C, 60.49; H, 3.40; N, 2.87.

*(E)-1-(4-Chlorophenyl)-3-[4-((7-chloroquinolin-4-yl)oxy)-3-methoxyphenyl]prop-2-en-1-one* (**5b**). White solid; 95% yield; mp: 168–170 °C. FTIR ʋ (cm^−1^): 1661 (C=O), 1603 and 1587 (C=C and C=N). ^1^H-NMR (CDCl_3_) δ ppm 3.82 (s, 3H, OCH_3_), 6.43 (d, *J* = 5.2 Hz, 1H, H-3), 7.22 (d, *J* = 8.2 Hz, 1H, H*o*'), 7.29 (d, *J* = 1.7 Hz, 1H, H*m*), 7.35 (dd, *J* = 8.2, 1.7 Hz, 1H, H*m*'), 7.45 (d, *J* = 15.8, 1H, =CH), 7.49 (d, *J* = 8.5 Hz, 2H, H*o*''), 7.53 (dd, *J* = 8.9, 2.0 Hz, 1H, H-6), 7.81 (d, *J* = 15.8, 1H, =CH), 7. 98 (d, *J* = 8.5 Hz, 2H, H*m*''), 8.09 (d, *J* = 2.0 Hz, 1H, H-8), 8.33 (d, *J* = 8.9 Hz, 1H, H-5), 8.64 (d, *J* = 5.2 Hz, 1H, H-2). ^13^C-NMR (CDCl_3_) δ ppm 56.0, 103.7, 112.7, 119.5, 122.0, 123.2, 123.5, 127.1, 128.0, 128.1, 128.7, 130.1, 132.0, 133.8, 136.2, 136.8, 144.2, 144.3, 150.2, 152.0, 152.2, 161.4, 189.2. MS (70 eV) *m/z* (%): 449 (100, M^+^), 414 (38), 271 (46), 160 (35), 139 (58), 111 (41). Anal. Calcd. For C_25_H_17_Cl_2_NO_3_: C, 66.68; H, 3.81; N, 3.11. Found: C, 66.35; H, 3.79; N, 3.07.

*(E)-3-[4-((7-Chloroquinolin-4-yl)oxy)-3-methoxyphenyl]-1-phenylprop-2-en-1-one* (**5c**). White solid; 89% yield; mp: 157–159 °C. FTIR ʋ (cm^−1^): 1661 (C=O), 1603 and 1583 (C=N and C=C). ^1^H-NMR (CDCl_3_) δ ppm 3.81 (s, 3H, OCH_3_), 6.43 (d, *J* = 5.3 Hz, 1H, H-3), 7.22 (d, *J* = 8.2 Hz, 1H, H*o*'), 7.30 (d, *J* = 1.6 Hz, 1H, H*m*), 7.34 (dd, *J* = 8.2, 1.6 Hz, 1H, H*m*'), 7.47–7.63 (m, 5H, =CH, H-6, H*o*'' and H*p*''), 7.80 (d, *J* = 15.6, 1H, =CH), 8.03 (d, *J* = 7.3 Hz, 2H, H*m*''), 8.08 (d, *J* = 1.8 Hz, 1H, H-8), 8.33 (d, *J* = 8.8 Hz, 1H, H-5), 8.64 (d, *J* = 5.3 Hz, 1H, H-2). ^13^C-NMR (CDCl_3_) δ ppm 56.0, 103.7, 112.6, 119.5, 122.0, 122.7, 123.2, 123.5, 127.1, 128.0, 128.6, 128.7, 133.0, 134.0, 136.1, 138.1, 143.8, 144.1, 150.2, 151.9, 152.2, 161.4, 190.3. MS (70 eV) *m/z* (%): 415 (100, M^+^), 313 (30), 237 (39), 176 (48), 160 (31), 105 (60), 77 (56). Anal. Calcd. For C_25_H_18_ClNO_3_: C, 72.20; H, 4.36; N, 3.37. Found: C, 72.01; H, 4.34; N, 3.39.

*(E)-3-[4-((7-Chloroquinolin-4-yl)oxy)-3-methoxyphenyl]-1-(4-methoxyphenyl)prop-2-en-1-one* (**5d**). White solid; 62% yield; mp: 190–192 °C. FTIR ʋ (cm^−1^): 1655 (C=O), 1606 (C=N and C=C). ^1^H-NMR (CDCl_3_) δ ppm 3.80 (s, 3H, OCH_3_-Ar.C), 3.88 (s, 3H, OCH_3_-Ar.A), 6.43 (d, *J* = 5.3 Hz, 1H, H-3), 6.98 (d, *J* = 8.9 Hz, 2H, H*o*''), 7.20 (d, *J* = 8.0 Hz, 1H, H*o*'), 7.28 (d, *J* = 1.6 Hz, 1H, H*m*), 7.33 (dd, *J* = 8.0, 1.6 Hz, 1H, H*m*'), 7.50–7.54 (m, 2H, =CH, H-6), 7.79 (d, *J* = 15.6, 1H, =CH), 8.04 (d, *J* = 8.9 Hz, 2H, H*m*''), 8.07 (d, *J* = 1.9 Hz, 1H, H-8), 8.33 (d, *J* = 8.9 Hz, 1H, H-5), 8.63 (d, *J* = 5.3 Hz, 1H, H-2). ^13^C-NMR (CDCl_3_) δ ppm 55.5, 56.0, 103.7, 112.6, 114.0, 119.5, 121.8, 122.5, 123.2, 123.5, 127.1, 128.0, 130.7, 131.1, 134.3, 136.1, 142.9, 143.9, 150.2, 151.9, 152.2, 161.4, 163.6, 188.4. MS (70 eV) *m/z* (%): 445 (13, M^+^), 313 (85), 176 (98), 135 (100). Anal. Calcd. For C_26_H_20_ClNO_4_: C, 70.03; H, 4.52; N, 3.14. Found: C, 70.00; H, 4.50; N, 3.18.

*(E)-3-[4-((7-Chloroquinolin-4-yl)oxy)-3-methoxyphenyl]-1-(3,4,5-trimethoxyphenyl)prop-2-en-1-one* (**5e**). White solid; 58% yield; mp: 200–202 °C. FTIR ʋ (cm^−1^): 1650 (C=O), 1586 (C=N and C=C). ^1^H-NMR (DMSO-*d*_6_) δ ppm 3.79 (s, 3H, OCH_3_-Ar.A), 3.83 (s, 3H, OCH_3_-Ar.C), 3.92 (s, 6H, OCH_3_ × 2-Ar.A), 6.55 (d, *J* = 5.3 Hz, 1H, H-3), 7.42 (d, *J* = 8.3 Hz, 1H, H*o*'), 7.45 (s, 2H, H*o*''), 7.68–7.84 (m, 4H, H-6, H*m*, H*m*', =CH), 7.98 (d, *J* = 15.8, 1H, =CH), 8.09 (d, *J* = 2.0 Hz, 1H, H-8), 8.37 (d, *J* = 8.9 Hz, 1H, H-5), 8.70 (d, *J* = 5.3 Hz, 1H, H-2). ^13^C-NMR (DMSO-*d*_6_) δ ppm 56.1, 56.5, 61.0, 103.6, 106.4, 113.0, 119.5, 121.6, 122.4, 123.2, 123.5, 127.1, 128.0, 133.3, 134.1, 136.1, 142.9, 143.6, 144.1, 150.2, 151.9, 152.2, 153.3, 161.4, 189.0. MS (70 eV) *m/z* (%): 505 (100, M^+^), 490 (97), 195 (63), 160 (32). Anal. Calcd. For C_28_H_24_ClNO_6_: C, 66.47; H, 4.78; N, 2.77. Found: C, 66.42; H, 4.71; N, 2.69.

*(E)-3-[4-((7-Chloroquinolin-4-yl)oxy)-3-methoxyphenyl]-1-(p-tolyl)prop-2-en-1-one* (**5f**). White solid; 85% yield; mp: 182–183 °C. FTIR ʋ (cm^−1^): 1658 (C=O), 1604 and 1564 (C=N and C=C). ^1^H-NMR (CDCl_3_) δ ppm 2.43 (s, 3H, CH_3_), 3.81 (s, 3H, OCH_3_), 6.43 (d, *J* = 5.3 Hz, 1H, H-3), 7.21 (d, *J* = 8.0 Hz, 1H, H*o*'), 7.29 (d, *J* = 2.0 Hz, 1H, H*m*), 7.30–7.34 (m, 3H, H*o*'', H*m*'), 7.47–7.54 (m, 2H, H-6, =CH), 7.79 (d, *J* = 15.6, 1H, =CH), 7.94 (d, *J* = 8.3 Hz, 2H, H*m*''), 8.08 (d, *J* = 1.8 Hz, 1H, H-8), 8.33 (d, *J* = 9.0 Hz, 1H, H-5), 8.63 (d, *J* = 5.3 Hz, 1H, H-2). ^13^C-NMR (CDCl_3_) δ ppm 21.7, 56.0, 103.7, 112.6, 119.5, 121.9, 122.7, 123.2, 123.5, 127.1, 128.0, 128.7, 129.4, 134.2, 135.5, 136.1, 143.3, 143.9, 144.0, 150.2, 151.9, 152.2, 161.4, 189.8. MS (70 eV) *m/z* (%): 429 (61, M^+^), 313 (76), 176 (100), 135 (32), 119 (64), 91 (58). Anal. Calcd. For C_26_H_20_ClNO_3_: C, 72.64; H, 4.69; N, 3.26. Found: C, 72.59; H, 4.59; N, 3.33.

#### 3.2.3. General Procedure for the Synthesis of the *NH*-Pyrazolines **6a**–**f**

A mixture of chalcone **5** (100 mg, 0.20 mmol), hydrazine hydrate (0.26 mmol) in absolute ethanol (15 mL) was heated under reflux for 1 h until complete consumption of the chalcone (TLC control). The solid formed was filtered and washed of cold ethanol/water (1:0.5) mixture. No further purification was required.

*4-(4-(3-(4-Bromophenyl)-4,5-dihydro-1H-pyrazol-5-yl)-2-methoxyphenoxy)-7-chloroquinoline* (**6a**). White solid; 96% yield; mp: 230–232 °C. FTIR ʋ (cm^−1^): 3331 (NH), 1611 and 1587 (C=N and C=C). ^1^H-NMR (DMSO-*d*_6_) δ ppm 2.95 (dd, *J* = 16.3, 11.2 Hz, 1H, H-4'a), 3.51 (dd, *J* = 16.3, 11.2 Hz, 1H, H-4'b), 3.73 (s, 3H, OCH_3_), 4.96 (m, 1H, H-5'), 6.44 (d, *J* = 5.3 Hz, 1H, H-3), 7.09 (dd, *J* = 8.3, 1.5 Hz, 1H, H*m*'), 7.29 (d, *J* = 8.3 Hz, 1H, H*o*'), 7.32 (d, *J* = 1.5 Hz, 1H, H*m*), 7.51–7.62 (m, 4H, H*o*'', H*m*''), 7.68 (dd, *J* = 8.9, 2.0 Hz, 1H, H-6), 8.07 (d, *J* = 2.0 Hz, 1H, H-8), 8.35 (d, *J* = 8.9 Hz, 1H, H-5), 8.68 (d, *J* = 5.3 Hz, 1H, H-2), Not observed (s, 1H, NH).^13^C-NMR (-*d*_6_) δ ppm 41.6, 56.8, 63.8, 104.6, 113.6, 119.7, 120.1, 122.7, 124.5, 126.1, 127.1, 127.9, 129.4, 131.2, 132.6, 135.2, 138.1, 141.6, 143.5, 149.1, 150.2, 151.2, 153.2. MS (70 eV) *m/z* (%): 507 (38, M^+^), 509 (100), 495 (65), 414 (31), 312 (81), 285 (29), 183 (62), 176 (46), 155 (32), 135 (39), 99 (29), 69 (52). Anal. Calcd. For C_25_H_19_BrClN_3_O_2_: C, 59.02; H, 3.76; N, 8.26. Found: C, 59.13; H, 3.80; N, 8.31.

*7-Chloro-4-(4-(3-(4-chlorophenyl)-4,5-dihydro-1H-pyrazol-5-yl)-2-methoxyphenoxy)quinoline* (**6b**). White solid; 95% yield; mp: 223-225 °C. FTIR ʋ (cm^−1^): 3333 (NH), 1611 and 1589 (C=N and C=C). ^1^H-NMR (DMSO-*d*_6_) δ ppm 2.97 (dd, *J* = 16.3, 10.8 Hz, 1H, H-4'a), 3.52 (dd, *J* = 16.3, 10.8 Hz, 1H, H-4'b), 3.74 (s, 3H, OCH_3_), 4.98 (m, 1H, H-5'), 6.49 (d, *J* = 5.1 Hz, 1H, H-3), 7.10 (dd, *J* = 8.0, 1.8 Hz, 1H, H*m*'), 7.26 (d, *J* = 8.0 Hz, 1H, H*o*'), 7.30 (d, *J* = 1.8 Hz, 1H, H*m*), 7.43 (d, *J* = 8.5 Hz, 2H, H*o*''), 7.56 (dd, *J* = 9.0, 2.0 Hz, 1H, H-6), 7.66 (d, *J* = 8.5 Hz, 2H, H*m*''), 8.05 (d, *J* = 2.0 Hz, 1H, H-8), 8.36 (d, *J* = 9.0 Hz, 1H, H-5), 8.68 (d, *J* = 5.1 Hz, 1H, H-2), Not observed (s, 1H, NH). ^13^C-NMR (DMSO-*d*_6_) δ ppm 41.4, 56.8, 65.2, 103.4, 112.2, 114.2, 119.1, 119.7, 120.3, 122.5, 124.3, 125.8, 126.0, 127.1, 127.4, 127.7, 128.0, 128.5, 129.2, 133.3, 134.0, 135.3, 153.1. MS (70 eV) *m/z* (%): 463 (92, M^+^), 449 (64), 312 (87), 298 (38), 285 (37), 271 (34), 177 (52), 162 (40), 151 (98), 139 (100), 111 (40). Anal. Calcd. For C_25_H_19_Cl_2_N_3_O_2_: C, 64.66; H, 4.12; N, 9.05. Found: C, 64.57; H, 4.09; N, 9.11.

*7-Chloro-4-(2-methoxy-4-(3-phenyl-4,5-dihydro-1H-pyrazol-5-yl)phenoxy)quinoline* (**6c**). White solid; 81% yield; mp: 178–179 °C. FTIR (ʋ (cm^−1^): 3345 (NH), 1612 and 1589 (C=N and C=C). ^1^H-NMR (DMSO-*d*_6_) δ ppm 2.99 (dd, *J* = 16.1, 10.7 Hz, 1H, H-4'a), 3.53 (dd, *J* = 16.1, 10.7 Hz, 1H, H-4'b), 3.75 (s, 3H, OCH_3_), 4.98 (m, 1H, H-5'), 6.51 (d, *J* = 4.9 Hz, 1H, H-3), 7.12 (d, *J* = 8.2 Hz, 1H, H*m*'), 7.24 (d, *J* = 8.2 Hz, 1H, H*o*'), 7.29–7.43 (m, 4H, H*m*, H*m*'', H*p*''), 7.59–7.70 (m, 3H, H*o*'', H-6), 8.05 (s, 1H, H-8), 8.36 (d, *J* = 8.8 Hz, 1H, H-5), 8.68 (d, *J* = 4.9 Hz, 1H, H-2), Not observed (s, 1H, NH). ^13^C-NMR (DMSO-*d*_6_) δ ppm 41.2, 56.7, 63.9, 104.4, 112.6, 113.2, 119.0, 119.7, 120.1, 122.9, 124.2, 125.8, 126.0, 127.1, 127.8, 127.9, 128.5, 128.8, 129.2, 133.3, 134.5, 135.4, 153.2. MS (70 eV) *m/z* (%): 431 (5, M^+^), 327 (64), 312 (100), 176 (40). Anal. Calcd. For C_25_H_20_ClN_3_O_2_: C, 69.85; H, 4.69; N, 9.77. Found: C, 69.71; H, 4.75; N, 9.69.

*7-Chloro-4-(2-methoxy-4-(3-(4-methoxyphenyl)-4,5-dihydro-1H-pyrazol-5-yl)phenoxy)quinoline* (**6d**). White solid; 76% yield; mp: 214–216 °C. FTIR ʋ (cm^−1^): 3215 (NH), 1568 and 1503 (C=N and C=C). ^1^H-NMR (DMSO-*d*_6_) δ ppm 2.91 (dd, *J* = 16.3, 10.9 Hz, 1H, H-4'a), 3.49 (dd, *J* = 16.3, 10.9 Hz, 1H, H-4'b), 3.73 (s, 3H, OCH_3_-Ar.C), 3.78 (s, 3H, OCH_3_-Ar.A), 4.90 (m, 1H, H-5'), 6.45 (d, *J* = 5.2 Hz, 1H, H-3), 6.96 (d, *J* = 8.8 Hz, 2H, H*o*''), 7.10 (dd, *J* = 8.2, 1.5 Hz, 1H, Hm'), 7.28 (d, *J* = 8.2 Hz, 1H, H*o*'), 7.33 (d, *J* = 1.5 Hz, 1H, H*m*), 7.59 (d, *J* = 8.8 Hz, 2H, H*m*''), 7.67 (dd, *J* = 8.9, 2.0 Hz, 1H, H-6), 8.07 (d, *J* = 2.0 Hz, 1H, H-8), 8.35 (d, *J* = 8.9 Hz, 1H, H-5), 8.68 (d, *J* = 5.2 Hz, 1H, H-2), Not observed (s, 1H, NH). ^13^C-NMR (DMSO-*d*_6_) δ ppm 40.5, 54.7, 55.3, 62.9, 103.1, 111.6, 113.5, 118.4, 119.0, 122.1, 123.4, 125.4, 126.3, 126.5, 126.9, 134.3, 139.7, 142.2, 148.5, 149.1, 150.5, 152.4, 158.9, 160.5. MS (70 eV) *m/z* (%): 459 (6, M^+^), 461 (8), 135 (100). Anal. Calcd. For C_26_H_22_ClN_3_O_3_: C, 67.90; H, 4.82; N, 9.14. Found: C, 67.85; H, 4.86; N, 9.09.

*7-Chloro-4-(2-methoxy-4-(3-(3,4,5-trimethoxyphenyl)-4,5-dihydro-1H-pyrazol-5-yl)phenoxy)-quinoline* (**6e**). White solid; 71% yield; mp: 226–227 °C. FTIR ʋ (cm^−1^): 3216 (NH), 1572 and 1507 (C=N and C=C). ^1^H-NMR (DMSO-*d*_6_) δ ppm Not observed (dd, 1H, H-4'a, overlapped with water signal), 3.53 (dd, *J* = 16.1, 10.7 Hz, 1H, H-4'b), 3.75 (s, 3H, OCH_3_-Ar.C), 3.76 (s, 3H, OCH_3_-Ar.A), 3.84 (s, 6H, OCH_3_ × 2-Ar.A), 4.96 (m, 1H, H-5'), 6.50 (d, *J* = 5.0 Hz, 1H, H-3), 6.97 (s, 2 H, H*o*''), 7.12 (d, *J* = 8.2 Hz, 1H, H*m*'), 7.25 (d, *J* = 8.2 Hz, 1H, H*o*'), 7.31 (s, 10H, H*m*), 7.65 (dd, *J* = 8.9, 1.4 Hz, 1H, H-6), 8.05 (d, *J* = 1.4 Hz, 1H, H-8), 8.36 (d, *J* = 8.9 Hz, 1H, H-5), 8.68 (d, *J* = 5.0 Hz, 1H, H-2), Not observed (s, 1H, NH). ^13^C-NMR (DMSO-*d*_6_) δ ppm 41.4, 56.7, 56.9, 63.9, 104.4, 104.8, 105.0, 113.3, 119.7, 120.0, 122.9, 124.2, 124.9, 127.1, 127.9, 129.0, 129.4, 137.8, 139.6, 141.6, 143.1, 149.4, 153.2, 153.6, 161.2. MS (70 eV) *m/z* (%): 519 (2, M^+^), 521 (8), 313 (62), 210 (55), 195 (100), 176 (66). Anal. Calcd. For C_28_H_26_ClN_3_O_5_: C, 64.68; H, 5.04; N, 8.08. Found: C, 64.59; H, 5.09; N, 8.13.

*7-Chloro-4-(2-methoxy-4-(3-(p-tolyl)-4,5-dihydro-1H-pyrazol-5-yl)phenoxy)quinoline* (**6f**). White solid; 80% yield; mp: 179–181 °C. FTIR ʋ (cm^−1^): 3335 (NH), 1601 and 1574 (C=N and C=C). ^1^H-NMR (DMSO-*d*_6_) δ ppm 2.33 (s, 3H, CH_3_), 2.95 (dd, *J* = 16.0, 10.5 Hz, 1H, H-4'a), 3.50 (dd, *J* = 16.0, 10.5 Hz, 1H, H-4'b), 3.74 (s, 3H, OCH_3_), 4.94 (m, 1H, H-5'), 6.50 (d, *J* = 4.6 Hz, 1H, H-3), 7.11 (d, *J* = 8.2 Hz, 1H, H*m*'), 7.20 (d, *J* = 7.5 Hz, 2H, H*o*''), 7.24 (d, *J* = 8.2 Hz, 1H, Ho'), 7.31 (s, 2H, H*m*, NH), 7.55 (d, *J* = 7.5 Hz, 2H, H*m*''), 7.65 (d, *J* = 8.8 Hz, 1H, H-6), 8.05 (s, 1H, H-8), 8.36 (d, *J* = 8.8 Hz, 1H, H-5), 8.68 (d, *J* = 4.6 Hz, 1H, H-2). ^13^C-NMR (DMSO-*d*_6_) δ ppm 21.2, 56.7, 63.8, 104.4, 113.3, 119.7, 120.0, 122.8, 124.2, 126.0, 127.1, 127.9, 129.4, 131.2, 132.6, 135.3, 138.1, 141.6, 143.1, 149.5, 150.3, 151.7, 153.2, 161.6. MS (70 eV) *m/z* (%): 443 (3, M^+^), 445 (24), 176 (42), 119 (100), 91 (58). Anal. Calcd. For C_26_H_22_ClN_3_O_2_: C, 70.34; H, 5.00; N, 9.47. Found: C, 70.07; H, 5.03; N, 9.50.

#### 3.2.4. General Procedure for the Synthesis of the *N*-Acetylpyrazolines **7a**–**f**

A mixture of the *NH*-pyrazoline **6** (50 mg, 0.10 mmol), acetic anhydride (2 mL) and pyridine (3 drops) was stirred at room temperature for 10 min until complete consumption of the *NH*-pyrazoline (TLC control). Then, water (3 mL) was added and the resulting precipitate was collected by filtration, washed with water and recrystallized from ethanol.

*1-(3-(4-Bromophenyl)-5-[4-(7-chloroquinolin-4-yloxy)-3-methoxyphenyl]-4,5-dihydro-1H-pyrazol-1-yl)ethanone* (**7a**). White solid; 91% yield; mp: 244–245 °C. FTIR ʋ (cm^−1^): 1665 (C=O), 1585 (C=N and C=C). ^1^H-NMR (DMSO-*d*_6_) δ ppm Not observed (dd, 1H, H-4'a, overlapped with water signal), 2.35 (s, 3H, CH_3_), 3.71 (s, 3H, OCH_3_), 3.91 (dd, *J* = 18.1, 11.8 Hz, 1H, H-4'b), 5.64 (dd, *J* = 11.8, 4.8 Hz, 1H, H-5'), 6.43 (d, *J* = 5.3 Hz, 1H, H-3), 6.85 (dd, *J* = 8.2, 1.8 Hz, 1H, H*m*'), 7.14 (d, *J* = 1.8 Hz, 1H, H*m*), 7.26 (d, *J* = 8.2 Hz, 1H, H*o*'), 7.65–7.71 (m, 3H, H-6, H*o*''), 7.73–7.77 (m, 2H, H*m*''), 8.07 (d, *J* = 2.0 Hz, 1H, H-8), 8.33 (d, *J* = 8.8 Hz, 1H, H-5), 8.67 (d, *J* = 5.3 Hz, 1H, H-2). ^13^C-NMR (DMSO-*d*_6_) δ ppm 21.9, 42.2, 56.0, 60.0, 100.0, 103.7, 111.4, 118.0, 119.0, 123.0, 123.3, 123.9, 127.0, 127.5, 128.8, 130.5, 131.9, 134.9, 140.4, 140.8, 149.7, 151.3, 153.0, 153.5, 167.9. MS (70 eV) *m/z* (%): 549 (34, M^+^), 551 (44), 509 (63), 368 (56), 285 (32), 43 (100). Anal. Calcd. For C_27_H_21_BrClN_3_O_3_: C, 58.87; H, 3.84; N, 7.63. Found: C, 58.35; H, 3.79; N, 7.70.

*1-(3-(4-Chlorophenyl)-5-[4-(7-chloroquinolin-4-yloxy)-3-methoxyphenyl]-4,5-dihydro-1H-pyrazol-1-yl)ethanone* (**7b**). White solid; 91% yield; mp: 236–238 °C. FTIR ʋ (cm^−1^): 1666 (C=O), 1595 (C=N and C=C). ^1^H-NMR (DMSO-*d*_6_) δ ppm 2.35 (s, 3H, CH_3_), 3.25 (dd, *J* = 17.8, 4.4 Hz, 1H, H-4'a), 3.72 (s, 3H, OCH_3_), 3.91 (dd, *J* = 17.8, 11.7 Hz, 1H, H-4'b), 5.65 (dd, *J* = 11.7, 4.4 Hz, 1H, H-5'), 6.47 (d, *J* = 4.5 Hz, 1H, H-3), 6.88 (d, *J* = 7.5 Hz, 1H, H*m*'), 7.13 (s, 1H, H*m*), 7.23 (d, *J* = 7.5 Hz, 1H, H*o*'), 7.52 (d, *J* = 8.0 Hz, 2H, H*o*''), 7.65 (d, *J* = 8.8 Hz, 1H, H-6), 7.81 (d, *J* = 8.0 Hz, 2H, H*m*''), 8.05 (s, 1H, H-8), 8.33 (d, *J* = 8.8 Hz, 1H, H-5), 8.67 (d, *J* = 4.5 Hz, 1H, H-2). ^13^C-NMR (DMSO-*d*_6_) δ ppm 22.1, 42.6, 56.7, 60.2, 104.4, 112.6, 118.8, 119.7, 123.1, 124.2, 127.1, 127.2, 127.9, 128.8, 129.3, 135.1, 135.4, 141.7, 142.0, 150.3, 151.8, 153.2, 153.6, 161.4, 168.2. MS (70 eV) *m/z* (%): 505 (33, M^+^), 463 (55), 368 (41), 285 (31), 179 (54), 43 (100). Anal. Calcd. For C_27_H_21_Cl_2_N_3_O_3_: C, 64.04; H, 4.18; N, 8.30. Found: C, 63.97; H, 4.00; N, 8.36.

*1-(5-[4-(7-Chloroquinolin-4-yloxy)-3-methoxyphenyl]-3-phenyl-4,5-dihydro-1H-pyrazol-1-yl)ethanone* (**7c**). White solid; 90% yield; mp: 240–241 °C. FTIR ʋ (cm^−1^): 1663 (C=O), 1597 and 1570 (C=N and C=C). ^1^H-NMR (DMSO-*d*_6_) δ ppm 2.36 (s, 3H, CH_3_), 3.20–3.30 (m, 1H, H-4'a), 3.72 (s, 3H, OCH_3_), 3.93 (dd, *J* = 16.6, 12.6 Hz, 1H, H-4'b), 5.61–5.67 (m, 1H, H-5'), 6.49 (s, 1H, H-3), 6.89 (d, *J* = 6.9 Hz, 1H, H*m*'), 7.13 (s, 1H, H*m*), 7.22 (d, *J* = 6.9 Hz, 1H, H*o*'), 7.48 (s, 3H, H*o*'', H*p*''), 7.64 (d, *J* = 7.7 Hz, 1H, H-6), 7.81 (s, 2H, H*m*''), 8.04 (s, 1H, H-8), 8.33 (d, *J* = 7.7 Hz, 1H, H-5), 8.67 (s, 1H, H-2). ^13^C-NMR (DMSO-*d*_6_) δ ppm 22.2, 42.8, 56.6, 60.4, 104.8, 112.9, 118.8, 120.1, 123.4, 124.6, 127.6, 127.8, 127.9, 128.4, 129.3, 135.2, 135.5, 141.6, 142.0, 151.0, 152.4, 153.2, 154.3, 161.4, 167.9. MS (70 eV) *m/z* (%): 471 (75, M^+^), 429 (100), 368 (57), 178 (31), 145 (82), 104 (41), 43 (67). Anal. Calcd. For C_27_H_22_ClN_3_O_3_: C, 68.71; H, 4.70; N, 8.90. Found: C, 68.40; H, 4.75; N, 8.91.

*1-(5-[4-(7-Chloroquinolin-4-yloxy)-3-methoxyphenyl]-3-(4-methoxyphenyl)-4,5-dihydro-1H-pyrazol-1-yl)ethanone* (**7d**). White solid; 75% yield; mp: 238–239 °C. FTIR ʋ (cm^−1^): 1663 (C=O), 1607 and 1576 (C=N and C=C). ^1^H-NMR (DMSO-*d*_6_) δ ppm 2.34 (s, 3H, CH_3_), 3.22 (dd, *J* = 17.8, 2.8 Hz, 1H, H-4'a), 3.72 (s, 3H, OCH_3_-Ar.C), 3.81–3.94 (m, 4H, OCH_3_-Ar.A, H-4'b), 5.58–5.64 (m, 1H, H-5'), 6.49 (d, *J* = 2.8 Hz, 1H, H-3), 6.88 (d, *J* = 7.4 Hz, 1H, H*m*'), 7.03 (d, *J* = 7.4 Hz, 2H, Ho''), 7.12 (s, 1H, H*m*), 7.22 (d, *J* = 7.4 Hz, 1H, H*o*') 7.64 (d, *J* = 8.2 Hz, 1H, H-6) 7.75 (d, *J* = 7.4 Hz, 2H, H*m*'') 8.04 (s, 1H, H-8) 8.33 (d, *J* = 8.2 Hz, 1H, H-5) 8.67 (d, *J* = 2.8 Hz, 1H, H-2). ^13^C-NMR (DMSO-*d*_6_) δ ppm 22.1, 42.8, 56.0, 56.7, 59.9, 100.2, 104.4, 112.5, 114.9, 118.7, 119.7, 123.1, 124.2, 124.5, 127.1, 127.9, 128.7, 135.3, 141.6, 142.3, 150.6, 151.8, 153.2, 154.4, 161.4, 168.3. MS (70 eV) *m/z* (%): 501 (74, M^+^), 459 (67), 175 (40), 134 (42), 43 (100). Anal. Calcd. For C_28_H_24_ClN_3_O_4_: C, 67.00; H, 4.82; N, 8.37. Found: C, 67.15; H, 4.87; N, 8.29.

*1-(5-[4-(7-Chloroquinolin-4-yloxy)-3-methoxyphenyl]-3-(3,4,5-trimethoxyphenyl)-4,5-dihydro-1H-pyrazol-1-yl)ethanone* (**7e**). White solid; 73% yield; mp: 223–224 °C. FTIR ʋ (cm^−1^): 1659 (C=O), 1603 and 1572 (C=N and C=C). ^1^H-NMR (DMSO-*d*_6_) δ ppm 2.36 (s, 3H, CH_3_) 3.28 (dd, *J* = 18.1, 4.0 Hz, 1H, H-4'a) 3.73 (s, 3H, OCH_3_-Ar.C) 3.76 (s, 3H, OCH_3_-Ar.A) 3.82–3.96 (m, 7H, OCH_3_ × 2-Ar.A and H-4'b) 5.64 (dd, *J* = 11.3, 4.0 Hz, 1H, H-5') 6.49 (d, *J* = 4.8 Hz, 1H, H-3) 6.89 (d, *J* = 8.2 Hz, 1H, H*m*') 7.06–7.16 (m, 3H, H*m* and H*o*'') 7.23 (d, *J* = 8.2 Hz, 1H, H*o*') 7.65 (d, *J* = 8.9 Hz, 1H, H-6) 8.05 (s, 1H, H-8) 8.34 (d, *J* = 8.9 Hz, 1H, H-5) 8.68 (d, *J* = 4.8 Hz, 1H, H-2). ^13^C-NMR (DMSO-*d*_6_) δ ppm 22.1, 42.8, 56.7, 57.1, 60.1, 60.7, 104.4, 106.0, 112.5, 118.7, 119.7, 123.1, 124.2, 127.1, 127.2, 127.9, 135.3, 141.2, 141.6, 142.2, 150.3, 151.8, 153.2, 153.8, 154.5, 161.5, 168.1. MS (70 eV) *m/z* (%): 561 (100, M^+^), 519 (73), 504 (32), 285 (19), 236 (36), 97 (33), 83 (36), 57 (53), 43 (52). Anal. Calcd. For C_30_H_28_ClN_3_O_6_: C, 64.11; H, 5.02; N, 7.48. Found: C, 64.38; H, 5.10; N, 7.56.

*1-(5-[4-(7-Chloroquinolin-4-yloxy)-3-methoxyphenyl]-3-(p-toluyl)-4,5-dihydro-1H-pyrazol-1-yl)-ethanone* (**7f**). White solid; 85% yield; mp: 250–251 °C. FTIR ʋ (cm^−1^): 1663 (C=O), 1601 and 1576 (C=N and C=C). ^1^H-NMR (DMSO-*d*_6_) δ ppm 2.35 (s, 3H, CH_3_-Ar.A), 2.37 (s, 3H, CH_3_-Ar.B) 3.22 (dd, *J* = 17.7, 3.7 Hz, 1H, H-4'a) 3.72 (s, 3H, OCH_3_) 3.90 (dd, *J* = 17.7, 9.6 Hz, 1H, H-4'b) 5.62 (dd, *J* = 9.6, 3.7 Hz, 1H, H-5') 6.49 (d, *J* = 4.4 Hz, 1H, H-3) 6.88 (d, *J* = 7.4 Hz, 1H, H*m*') 7.12 (s, 1H, H*m*) 7.22 (d, *J* = 7.4 Hz, 1H, H*o*') 7.29 (d, *J* = 6.9 Hz, 2H, H*o*'') 7.65 (d, *J* = 8.4 Hz, 1H, H-6) 7.70 (d, *J* = 6.9 Hz, 2H, H*m*'') 8.04 (s, 1H, H-8) 8.34 (d, *J* = 8.4 Hz, 1H, H-5) 8.67 (d, *J* = 4.4 Hz, 1H, H-2). ^13^C-NMR (DMSO-*d*_6_) δ ppm 21.3, 22.1, 42.7, 56.7, 59.9, 104.5, 112.5, 118.7, 119.7, 123.1, 124.2, 126.6, 127.3, 127.9, 128.6, 129.4, 135.3, 140.6, 141.6, 142.2, 150.3, 151.8, 153.2, 154.6, 161.4, 168.1. MS (70 eV) *m/z* (%): 485 (86, M^+^), 443 (100), 159 (68), 118 (43), 91 (29), 43 (44). Anal. Calcd. For C_28_H_24_ClN_3_O_3_: C, 69.20; H, 4.98; N, 8.65. Found: C, 69.27; H, 4.88; N, 8.31.

#### 3.2.5. General Procedure for the Synthesis of the *N*-Formylpyrazolines **8a**–**f**

A mixture of the *NH*-pyrazoline **6** (50 mg, 0.10 mmol) and formic acid (2 mL) was stirred at room temperature for 30 min until complete consumption of the *NH*-pyrazoline (TLC control). Then, the adding of crushed ice to the solution precipitated a solid which was filtered, washed with water and recrystallized from ethanol.

*3-(4-Bromophenyl)-5-[4-(7-chloroquinolin-4-yloxy)-3-methoxyphenyl]-4,5-dihydro-1H-pyrazole-1-carbaldehyde* (**8a**). White solid; 94% yield; mp: 230–231 °C. FTIR ʋ (cm^−1^): 1674 (C=O), 1593 (C=N and C=C). ^1^H-NMR (DMSO-*d*_6_) δ ppm 3.28–3.36 (m, 1H, H-4'a), 3.73 (s, 3H, OCH_3_), 3.91–4.03 (m, 1H, H-4'b), 5.60–5.66 (m, 1H, H-5'), 6.50 (d, *J* = 1.9 Hz, 1H, H-3), 6.96 (d, *J* = 6.7 Hz, 1H, H*m*'), 7.18 (s, 1H, H*m*), 7.25 (d, *J* = 6.7 Hz, 1H, H*o*'), 7.59–7.82 (m, 5H, H*m*'', H-6, H*o*''), 8.05 (s, 1H, H-8), 8.34 (d, *J* = 8.3 Hz, 1H, H-5), 8.68 (d, *J* = 1.9 Hz, 1H, H-2), 8.95 (s, 1H, CHO). ^13^C-NMR (DMSO-*d*_6_) δ ppm 42.2, 55.8, 58.6, 99.5, 103.6, 111.4, 118.3, 118.8, 122.9, 123.8, 124.0, 126.8, 127.4, 128.7, 131.8, 134.8, 137.4, 140.5, 150.3, 151.2, 152.9, 155.3, 159.9, 160.8. MS (70 eV) *m/z* (%): 535 (47, M^+^), 537 (64), 354 (25), 197 (30), 176 (92), 85 (66), 83 (100), 47 (33). Anal. Calcd. For C_26_H_19_BrClN_3_O_3_: C, 58.17; H, 3.57; N, 7.83. Found: C, 58.09; H, 3.55; N, 7.87.

*3-(4-Chlorophenyl)-5-[4-(7-chloroquinolin-4-yloxy)-3-methoxyphenyl]-4,5-dihydro-1H-pyrazole-1-carbaldehyde* (**8b**). White solid; 92% yield; mp: 206–209 °C. FTIR ʋ (cm^−1^): 1674 (C=O), 1591 (C=N and C=C). ^1^H-NMR (DMSO-*d*_6_) δ ppm 3.33 (dd, *J* = 18.2, 5.1 Hz, 1H, H-4'a), 3.73 (s, 3H, OCH_3_), 3.98 (dd, *J* = 18.2, 11.6 Hz, 1H, H-4'b), 5.64 (dd, *J* = 11.6, 5.1 Hz, 1H, H-5'), 6.49 (d, *J* = 5.0 Hz, 1H, H-3), 6.95 (d, *J* = 8.0 Hz, 1H, H*m*'), 7.18 (s, 1H, H*m*), 7.25 (d, *J* = 8.0 Hz, 1H, H*o*'), 7.53 (d, *J* = 8.3 Hz, 2H, H*o*''), 7.65 (d, *J* = 8.8 Hz, 1H, H-6), 7.82 (d, *J* = 8.3 Hz, 2H, H*m*''), 8.05 (s, 1H, H-8), 8.34 (d, *J* = 8.8 Hz, 1H, H-5), 8.67 (d, *J* = 5.0 Hz, 1H, H-2), 8.94 (s. 1H, CHO). ^13^C-NMR (DMSO-*d*_6_) δ ppm 42.3, 55.9, 58.6, 99.5, 103.6, 111.5, 118.2, 118.8, 122.9, 123.8, 126.8, 127.4, 128.5, 128.9, 129.7, 134.8, 135.2, 140.5, 149.5, 151.2, 152.9, 155.2, 160.0, 160.8. MS (70 eV) *m/z* (%): 491 (95, M^+^), 311 (33), 176 (100), 153 (61), 138 (54), 85 (38), 83 (59), 43 (25). Anal. Calcd. For C_26_H_19_Cl_2_N_3_O_3_: C, 63.43; H, 3.89; N, 8.53. Found: C, 63.11; H, 3.96; N, 8.42.

*5-[4-(7-Chloroquinolin-4-yloxy)-3-methoxyphenyl]-3-phenyl-4,5-dihydro-1H-pyrazole-1-carbaldehyde* (**8c**). White solid; 88% yield; mp: 208–210 °C. FTIR ʋ (cm^−1^): 1676 (C=O), 1585 (C=N and C=C). ^1^H-NMR (DMSO-*d*_6_) δ ppm Not observed (dd, 1H, H-4'a, overlapped with water signal), 3.72 (s, 3H, OCH_3_), 3.99 (dd, *J* = 18.1, 11.8 Hz, 1H, H-4'b), 5.64 (dd, *J* = 11.8, 5.0 Hz, 1H, H-5'), 6.44 (d, *J* = 5.3 Hz, 1H, H-3), 6.92 (dd, *J* = 8.2, 1.8 Hz, 1H, H*m*'), 7.19 (d, *J* = 1.8 Hz, 1H, H*m*), 7.28 (d, *J* = 8.2 Hz, 1H, H*o*'), 7.46–7.53 (m, 3H, H*o*'', H*p*''), 7.67 (dd, *J* = 8.8, 2.1 Hz, 1H, H-6), 7.79–7.85 (m, 2H, H*m*''), 8.07 (d, *J* = 2.1 Hz, 1H, H-8), 8.34 (d, *J* = 8.8 Hz, 1H, H-5), 8.67 (d, *J* = 5.3 Hz, 1H, H-2), 8.97 (s, 1H, CHO). ^13^C-NMR (DMSO-*d*_6_) δ ppm 41.6, 55.5, 57.9, 103.2, 111.5, 117.9, 118.4, 122.0, 122.9, 126.0, 126.7, 127.9, 130.0, 130.2, 134.1, 139.7, 140.7, 140.5, 149.0, 150.7, 151.9, 155.4, 159.9, 160.2. MS (70 eV) *m/z* (%): 457 (100, M^+^), 176 (72), 145 (59), 119 (65), 104 (55). Anal. Calcd. For C_26_H_20_ClN_3_O_3_: C, 68.20; H, 4.40; N, 9.18. Found: C, 68.12; H, 4.52; N, 9.03.

*5-[4-(7-Chloroquinolin-4-yloxy)-3-methoxyphenyl]-3-(4-methoxyphenyl)-4,5-dihydro-1H-pyrazole-1-carbaldehyde* (**8d**). White solid; 86% yield; mp: 239–240 °C. FTIR ʋ (cm^−1^): 1670 (C=O), 1603 (C=N and C=C). ^1^H-NMR (DMSO-*d*_6_) δ ppm Not observed (dd, 1H, H-4'a, overlapped with water signal), 3.71 (s, 3H, OCH_3_-Ar.C ) 3.81 (s, 3H, OCH_3_-Ar.A) 3.94 (dd, *J* = 17.9, 11.7 Hz, 1H, H-4'b) 5.60 (dd, *J* =11.7, 4.1 Hz, 1H, H-5') 6.44 (d, *J* = 4.9 Hz, 1H, H-3) 6.90 (d, *J* =8.0 Hz, 1H, H*m*') 7.03 (d, *J* = 8.3 Hz, 2H, H*o*'') 7.18 (s, 1H, H*m*) 7.27 (d, *J* = 8.0 Hz, 1H, H*o*') 7.66 (d, *J* = 8.5 Hz, 1H, H-6) 7.75 (d, *J* = 8.3 Hz, 2H, Hm'') 8.06 (s, 1H, H-8) 8.33 (d, *J* = 8.5 Hz, 1H, H-5) 8.67 (d, *J* = 4.9 Hz, 1H, H-2) 8.93 (s, 1H, CHO). ^13^C-NMR (DMSO-*d*_6_) δ ppm 42.4, 55.4, 55.9, 58.2, 103.6, 111.4, 114.3, 118.1, 118.8, 122.9, 123.8, 126.0, 126.8, 127.4, 128.4, 129.7, 134.8, 139.7, 140.8, 149.6, 150.7, 152.9, 155.9, 159.7, 161.2. MS (70 eV) *m/z* (%): 487 (100, M^+^), 175 (70), 149 (87), 134 (99). Anal. Calcd. For C_27_H_22_ClN_3_O_4_: C, 66.46; H, 4.54; N, 8.61. Found: C, 66.26; H, 4.56; N, 8.58.

*5-[4-(7-Chloroquinolin-4-yloxy)-3-methoxyphenyl]-3-(3,4,5-trimethoxyphenyl)-4,5-dihydro-1H-pyrazole-1-carbaldehyde* (**8e**). White solid; 83% yield; mp: 241–243 °C. FTIR ʋ (cm^−1^): 1674 (C=O), 1593 (C=N and C=C), 1036. ^1^H-NMR (DMSO-*d*_6_) δ ppm 3.38 (dd, *J* = 18.1, 5.0 Hz, 1H, H-4'a), 3.71 (s, 3H, OCH_3_-Ar.C), 3.73 (s, 3H, OCH_3_-Ar.A), 3.84 (s, 6H, OCH_3_x2-Ar.A), 3.96 (dd, *J* = 18.1, 11.8 Hz, 1H, H-4'b), 5.64 (dd, *J* = 11.8, 5.0 Hz, 1H, H-5'), 6.44 (d, *J* = 5.3 Hz, 1H, H-3), 6.91 (dd, *J* = 8.3, 1.3 Hz, 1H, H*m*'), 7.09 (s, 2H, H*o*''), 7.18 (d, *J* = 1.3 Hz, 1H, H*m*), 7.30 (d, *J* = 8.3 Hz, 1H, H*o*'), 7.67 (dd, *J* = 9.0, 1.8 Hz, 1H, H-6), 8.07 (d, *J* = 1.8 Hz, 1H, H-8), 8.34 (d, *J* = 9.0 Hz, 1H, H-5), 8.68 (d, *J* = 5.3 Hz, 1H, H-2), 8.97 (s, 1H, CHO). ^13^C-NMR (DMSO-*d*_6_) δ ppm 42.5, 55.9 56.1, 58.4, 60.2, 103.5, 104.3, 111.3, 118.1, 118.8, 123.0, 123.8, 126.1, 126.8, 127.4, 134.8, 139.7, 140.4, 140.7, 149.5, 151.2, 152.8, 153.1, 156.1, 159.8, 160.8. MS (70 eV) *m/z* (%): 561 (1, M^+^), 547 (100). Anal. Calcd. For C_29_H_26_ClN_3_O_6_: C, 63.56; H, 4.78; N, 7.67. Found: C, 63.32; H, 4.48 N, 7.61.

*5-[4-(7-Chloroquinolin-4-yloxy)-3-methoxyphenyl]-3-(p-toluyl)-4,5-dihydro-1H-pyrazole-1-carbaldehyde* (**8f**). White solid; 86% yield; mp: 233–235 °C. FTIR ʋ (cm^−1^): 1668 (C=O), 1601 (C=N and C=C). ^1^H-NMR (DMSO-*d*_6_) δ ppm 2.37 (s, 3H, CH_3_), Not observed (dd, 1H, H-4'a, overlapped with water signal), 3.72 (s, 3H, OCH_3_), 3.96 (dd, *J* = 18.1, 11.7 Hz, 1H, H-4'b), 5.62 (dd, *J* = 11.7, 4.8 Hz, 1H, H-5'), 6.45 (d, *J* = 5.2 Hz, 1H, H-3), 6.91 (d, *J* = 6.5 Hz, 1H, H*m*'), 7.18 (s, 1H, H*m*), 7.24–7.36 (m, 3H, Ho', H*o*''), 7.61 - 7.76 (m, 3H, H-6, Hm''), 8.07 (d, *J* = 2.0 Hz, 1H, H-8), 8.34 (d, *J* = 9.0 Hz, 1H, H-5), 8.68 (d, *J* = 5.2 Hz, 1H, H-2), 8.95 (s, 1H, CHO). ^13^C-NMR (DMSO-*d*_6_) δ ppm 21.0, 42.4, 55.9, 58.3, 103.6, 111.4, 117.8, 118.2, 122.9, 123.8, 126.7, 126.8, 127.4, 128.0, 128.6, 129.4, 132.4, 134.2, 135.6, 140.5, 149.8, 152.9, 155.6, 159.8, 160.9. MS (70 eV) *m/z* (%): 485 (1, M^+^), 471(100). Anal. Calcd. For C_27_H_22_ClN_3_O_3_: C, 68.71; H, 4.70; N, 8.90. Found: C, 68.66; H, 4.78; N, 8.93.

### 3.3. Anticancer Activity

The human tumor cell lines of the cancer screening panel were grown in RPMI 1640 medium containing 5% fetal bovine serum and 2 mM l-glutamine. For a typical screening experiment, cells are inoculated into 96 well microtiter plates. After cell inoculation, the microtiter plates were incubated at 37 °C, 5% CO_2_, 95% air and 100% relative humidity for 24 h prior to addition of tested compounds. After 24 h, two plates of each cell line were fixed *in situ* with TCA, to represent a measurement of the cell population for each cell line at the time of sample addition (T_z_). The samples were solubilized in dimethyl sulfoxide (DMSO) at 400-fold the desired final maximum test concentration and stored frozen prior to use. At the time of compounds addition, an aliquot of frozen concentrate was thawed and diluted to twice, the desired final maximum test concentration with complete medium containing 50 µg/mL gentamicin. Additional four, 10-fold or ½ log serial dilutions were made to provide a total of five drug concentrations plus control. Aliquots of 100 µL of these different sample dilutions were added to the appropriate microtiter wells already containing 100 µL of medium, resulting in the required final sample concentrations [30]. After the tested compounds were added, the plates were incubated for an additional 48 h at 37 °C, 5% CO_2_, 95% air, and 100% relative humidity. For adherent cells, the assay was terminated by the addition of cold TCA. Cells were fixed *in situ* by the gentle addition of 50 µL of cold 50% (w/v) TCA (final concentration, 10% TCA) and incubated for 60 min at 4 °C. The supernatant was discarded, and plates were washed five times with tap water and air dried. Sulforhodamine B (SRB) solution (100 µL) at 0.4% (w/v) in 1% acetic acid was added to each well, and plates were incubated for 10 min at room temperature. After staining, unbound dye was removed by washing five times with 1% acetic acid and the plates were air dried. Bound stain was subsequently solubilized with 10 mM trizma base, and the absorbance was read on an automated plate reader at a wavelength of 515 nm. Using the seven absorbance measurements [time zero (T_z_), control growth in the absence of drug (C), and test growth in the presence of drug at the five concentration levels (T_i_)], the percentage growth was calculated at each of the drug concentrations levels. Percentage growth inhibition was calculated as: [(T_i_ − T_Z_)/(C − T_Z_)] × 100 for concentrations for which T_i_ > T_z_, and [(T_i_ − T_Z_)/T_Z_] × 100 for concentrations for which T_i_ < T_z_. Three dose response parameters were calculated for each compound. Growth inhibition of 50% (GI_50_) was calculated from [(T_i_ − T_Z_)/(C − T_Z_)] × 100 = 50, which is the drug concentration resulting in a 50% lower net protein increase in the treated cells (measured by SRB staining) as compared to the net protein increase seen in the control cells. The drug concentration resulting in total growth inhibition (TGI) was calculated from Ti = Tz. The LC_50_ (concentration of drug resulting in a 50% reduction in the measured protein at the end of the drug treatment as compared to that at the beginning) indicating a net loss of cells following treatment was calculated from [(T_i_ − T_Z_)/T_Z_] × 100 = −50. Values were calculated for each of these three parameters if the level of activity is reached; however, if the effect was not reached or was exceeded, the value for that parameter was expressed as greater or less than the maximum or minimum concentration tested [30,31,32,33].

## 4. Conclusions

Novel series of *NH* **6a**–**f**, *N*-acetyl **7a**–**f** and *N*-formyl **8a**–**f** pyrazoline derivates were synthesized starting from chalcones **5** bearing a quinoline motive in their structures, through an interesting synthetic methodology, affording those products in acceptable to excellent yields and in short reaction times. The antitumor evaluation data revealed that derivatives **7b**,**d**,**f** and **8c**,**f** exhibited remarkable activity with GI_50_ values in the range from 10^−7^ to 10^−6^ M against different cancer cell lines. Owing to the results obtained, chemical studies are being conducted to improve the antitumor activities of such compounds.
